# Studies on Host–Parasite Relationship Between Soybean Plants and *Aphelenchoides besseyi*

**DOI:** 10.3390/life15071154

**Published:** 2025-07-21

**Authors:** Neveen Atta Elhamouly, Nehal Atta, Shiming Liu, Deliang Peng

**Affiliations:** 1 State Key Laboratory for Biology of Plant Diseases and Insect Pests, Institute of Plant Protection, Chinese Academy of Agricultural Sciences, Beijing 100193, China; neven.atta001@agr.menofia.edu.eg (N.A.E.); liushiming01@caas.cn (S.L.); 2 Department of Botany, Faculty of Agriculture, Menoufia University, Shibin El-Kom 32514, Egypt; 3 Genetics Department, Faculty of Agriculture, Menoufia University, Shibin El-Kom 32514, Egypt; nehal.ata@agr.menofia.edu.eg; 4 Shaanxi Key Laboratory of Plant Nematology, Bio-Agriculture Institute of Shaanxi, Xi’an 710043, China

**Keywords:** China, *Glycine max*, GSFR syndrome, *Aphelenchoides besseyi*, host–parasite relationship, phenotype, seed transmission

## Abstract

*Aphelenchoides besseyi* is considered a highly prevalent facultative plant-parasitic nematode and has a significant impact on various economically important crops globally. Due to the lack of knowledge on the efficacy of various management techniques, *A. besseyi* is still challenging to control in the open field. The present investigation successfully shed light on some significant new points, including the following: (1) *A. besseyi* was confirmed inside all soybean tissues—including roots, stems, leaves, and seeds—indicating its endoparasitic nature and its strong ability to reach the upper foliar system where it causes green stem and foliar retention syndrome (GSFR) symptoms; (2) inoculated plants exhibited reduced vegetative growth parameters, as non-inoculated control soybean plants showed higher values of plant height (PH), fresh root weight (FRW), and fresh shoot weight (FSW) compared to inoculated plants; (3) Yudou 29 was identified as highly resistant to *A. besseyi*, as results from the resistance screening assay among different Chinese soybean cultivars confirmed its strong resistance under natural field infestation conditions; and (4) soybean seeds may act as inoculum sources of *A. besseyi,* highlighting the need to develop more effective control measures to prevent or limit nematode dissemination through seed transmission.

## 1. Introduction

Soybean (*Glycine max* L.), sometimes referred to as the king of beans, is well recognized as a significant provider of both direct and indirect protein for human consumption globally. Furthermore, it provides animals with an important supply of protein [1]. When compared to other legume crops, it ranks second in terms of oil content (18–22%) and has the greatest protein level (40–42%) of all other legume crops [2]. Additionally, it is employed in the production of biofuel and aquaculture [3]. The global production of essential food items is heavily reliant on fifteen crops, one of which is soybean, which holds the fourth position in terms of total production and the fourth position in terms of planted area [4,5,6]. Although soybeans are regarded to be a self-pollinating crop, an extremely small amount of cross-pollination does take place when they are grown in natural environments [7]. Challenges related to emerging pests and diseases have become more severe as a result of an unsustainable cultivation system that includes monocultures, intense planting, and crop expansion into recently reclaimed cultivation regions. Plant-parasitic nematodes (PPNs) are considered one of the critical challenges that restrict the production of soybean globally, with approximately 10–15% yield losses annually, which may exceed 30–100% depending on several factors, including different nematode species, cultivar susceptibility, cultivation systems, prevailing temperatures, geographical location, and soil characteristics [8].

The presence of *Aphelenchoides besseyi* in soybean plants results in the development of green stem and foliar retention (GSFR) syndrome symptoms, which include dark-green coloration, blistering, distorted leaves, the infested fields remaining green, twisting with enlarged nodes, and significantly high levels of flower and pod abortion, which creates various challenges, especially for severe infections, which may cause a 100% loss of soybean production [9,10,11]. For a long time, the occurrence of the stay-green (Zhengqing) syndrome has been reported in the major soybean production fields in the Huang–Huai–Hai region of China [12]. The stay-green syndrome’s symptoms look like GSFR syndrome symptoms, which are characterized by the affected soybean plants having significant levels of pod production without seeds, and all the foliar system remains green for a prolonged period until maturation [11]. The authors have suggested that the causal origins of the stay-green syndrome may be climate abnormalities, soil nutrient imbalance, or the synergistic infection of virus and insects, especially stinkbugs [12].

According to [13], the quality of soybean seeds is reduced when the infected green ones are harvested along with the healthy seeds, which eventually could lead to huge damage to harvest machines. Interestingly, *Aphelenchoides* species exhibit variations in their life cycle, which distinguishes them from the majority of plant-parasitic nematodes (PPNs) [14]. They are characterized by a great ability to survive as facultative ecto- and endoparasite organisms by consuming saprophytic fungi, which allows them to persist in the absence of the host plants [13,15]. It is remarkable to note that in some crops like rice and common beans, *A. besseyi* can reach the upper foliar system by moving ectoparasitically [13,16], whereas, on other crops such as soybeans, they can penetrate the host plant endoparasitically via cortical cells, and in the migratory stages, they have the ability to enter the root and hypocotyls of the soybean plants [15,17]. Although [15] reported the presence of *A. besseyi* within xylem vessels, it remains unclear how much xylem vessels affect nematode mobility inside plants and whether or not this affects the development of the green stem and foliar retention syndrome on soybeans. Four days after inoculation, the majority of the plant’s aboveground structures (leaves, petioles, stems, and nodes) were infested with nematodes [15]. *A. besseyi* has an exceptional ability to persist in anhydrobiosis for many years on rice grains due to its ability to transmit via seeds [16,18]. Because PPNs have a great ability to cause significant yield losses in agricultural systems, particularly when they are not managed sustainably, researchers need to gather a lot of information related to nematode occurrence, density, crop damage level, and innovative strategies for managing and monitoring these nematode populations in areas before crop cultivation. Unfortunately, due to the lack of knowledge on the efficacy of various management techniques and the lack of sources of genetic resistance to *A. besseyi* in soybean cultivars, *A. besseyi* is still challenging to control in the open field. Recently, there have been significant efforts to develop methods for phenotyping and identifying cultivars resistant to *A. besseyi* [13].

Thus, the objective of the current research is to evaluate the resistance level of 17 different Chinese soybean cultivars against *A. besseyi* infection under controlled greenhouse and natural infested-field conditions described by [1], evaluate the capability of *A. besseyi* nematodes to penetrate soybean root systems and their movement through different inner connective tissues until they reach the upper foliar system parts of soybean plants [2], confirm the chronological progression of green stem and foliar retention (GSFR) syndrome symptoms from the first day post-inoculation (dpi) and its influences on the different vegetative growth parameters on the evaluated soybean cultivars under study [3], and determine if the soybean seeds can play a significant role as a source of inoculum for nematode dissemination.

## 2. Materials and Methods

### 2.1. Nematode Populations and Inoculum Preparation

*Aphelenchoides besseyi sensu stricto* populations were isolated from green stem and foliar retention (GSFR)-symptomatic soybean plants collected from various locations across China [19]. This population was identified morphologically, morphometrically, and genetically using its ITS region, 18S rRNA, and *COI* gene sequences [11]. One of the most important morphometric features is the post-uterine sac (PUS) length, which in our population was 27% of the total vulva–anus distance [20]. Sequences from the 18S rRNA region of our *A. besseyi* population showed homologies of 97.35% and 94.55% when aligned with *A. besseyi* populations provided by [10,21], respectively. *A. besseyi* nematodes were sub-cultured on *Fusarium oxysporum* using potato dextrose agar (PDA) media. They were then maintained in the darkness for 2 weeks at 25 ± 1 °C in a Bio-Oxygen Demand incubator. Subsequently, the nematode suspension was collected by rinsing accumulated water droplets from the Petri dish covers with ddH_2_O. Using a light microscope and a Peters slide, the final nematode concentration was adjusted to 1000 nematodes/mL. The suspension was then stored in a fridge at 5 °C until it was used in subsequent experiments [10].

### 2.2. Penetration Study Experiment Under Controlled Greenhouse Conditions

This experiment was conducted to confirm the root penetration of *A. besseyi* and its presence within the various upper tissues of soybean plants. Assessments were performed at eleven different intervals, starting from the 1st to 45th day post-inoculation (dpi), with measurements taken every three days. Seventeen different soybean cultivars were used, each initially inoculated with 1000 nematodes per plant (Table S1). In order to maintain the average relative humidity at 89.4% throughout the study, inoculated soybean plants were sprayed with a water mist for one minute every two hours during the day and every four hours during the night. These conditions were provided by [17], who recommended using a growth chamber to cultivate soybean plants under controlled conditions that mimic the essential environment for the GSFR syndrome development. To minimize the impact of AC airflow variability in the greenhouse, experiments were conducted using a completely randomized block design with three blocks, each containing pots of the 17 different soybean cultivars. Randomization was achieved by distributing the order of the cultivar pots randomly across the established blocks. All cultivars were inoculated simultaneously, and the entire experiment was repeated thrice. At each sampling timepoint, examined soybean seedlings were divided into two distinct groups, one for nematode extraction and the other for nematode staining. Non-inoculated seedlings served as negative controls and were maintained and sampled on the same days post-inoculation (dpis) as the inoculated treatments. The present study also included the assessment of some vegetative characteristics, such as plant height (PH), fresh root weight (FRW), and fresh shoot weight (FSW). FRW was determined by separating the roots of each soybean plant from the upper shoot portions, rinsing them with faucet water to remove any remaining soil, drying them on absorbent paper, and then weighing them. Subsequently, roots were stained with acid fuchsin dye and examined at 100x magnification using a light microscope, in accordance with [22].

Additionally, to determine PH and FSW, the same procedure described for the root system was followed with a few modifications, primarily immersing the entire shoot system in 100% methanol before staining with acid fuchsin. Finally, to assess the subsequent syndrome symptoms caused by *A. besseyi*, along with evaluating the vegetative characteristics (FRW, FSW, and PH), seventeen different soybean cultivars were artificially infected with *A. besseyi*, with seven replications for each cultivar, and another seven non-inoculated cultivars served as negative controls. All plants were then maintained under greenhouse conditions for two months.

### 2.3. Nematode Transmission by Soybean Seeds

Soybean seeds from 17 different commercial Chinese cultivars, characterized by their high yield production (Table S1), were immersed for 12 h in a 12 mL nematode suspension with a *Pi* of 500 nematodes seed^−1^. After that, they were cultivated in white 25 mm diameter rigid PVC plastic pipes containing sterilized soil and preserved under controlled greenhouse conditions for three weeks. Eight replicates were employed for each cultivar, and another eight non-inoculated plants served as the negative control. The entire experiment was repeated thrice. Finally, we calculated the seed infection rate and seed germination percentage for each cultivar.

$$Seed\;Infection\;Rate\;(\%)=\left[\frac{Number\;of\;infected\;seeds}{Total\;number\;of\;seeds}\right]*100$$

$$Seed\;Infection\;Rate\;(\%)=\left[\frac{Number\;of\;infected\;seeds}{Total\;number\;of\;seeds}\right]*100$$

### 2.4. Field Experiment

This experiment was conducted over two successive seasons in 2023 and 2024 at Langfang base, Hebei province, China (116.6065879 Longitude; 39.5110141 Latitude) to confirm the pathogenicity of *A. besseyi* and to screen the resistance response for 17 different Chinese soybean cultivars (Table S1). The study was carried out in one-meter-square plots, each containing two soybean rows separated by 0.7 m, with 18–23 plants per row. We evaluated four different inoculation periods (2 weeks and 3 weeks after emergence and the V5–V6 and V6–V7 vegetative stages) to determine the optimal infection stage for the nematode. Soybean cultivars were inoculated with 1000 nematodes per plant, applied in proximity to the root zone. Non-inoculated plants served as the negative control. Five replicates were employed for each trial, and the entire experiment was arranged in a completely randomized block design. The experimental field was characterized by moderate to high rainfall conditions and air temperatures ranging between 30 and 35 °C. The experimental soil had a pH of 7.83, and a drip irrigation system was used throughout the study. In accordance with [10,23], nematodes were extracted from the aboveground shoot system of each soybean cultivar and then quantified using a Peters slide and a light microscope. Furthermore, the total quantity of pods produced per plant was determined and compared to the non-inoculated negative control plants.

Currently, there is no standard diagrammatic scale available for assessing soybean cultivar responses to *A. besseyi* infestation. Since *A. besseyi* is a non-cyst-forming nematode, we could not use the traditional female index formula for screening resistance among different soybean cultivars. To gain a clear understanding of each cultivar’s response to *A. besseyi* infestation, we extracted the final nematode populations separately from each soybean organ (stems, leaves, pods, and seeds). Then, we calculated the average reproduction factor [RF = *Pf*/*Pi*, where *Pf* = final population, *Pi* = initial population] for this nematode on each cultivar alone. Additionally, we designed a new scale for the relative susceptibility index [RS = (*Pf* of the evaluated cultivar/*Pf* on the standard susceptible cultivar) * 100]. First, the obtained average reproduction values allowed us to differentiate between the different soybean cultivars evaluated according to their susceptibility to nematode infestation. The cultivar with the lowest RF value indicates the highest resistance to *A. besseyi* infection among all evaluated cultivars. Second, the results from the relative susceptibility scores enabled us to successfully design a new RS index scale, which can differentiate the evaluated soybean cultivars into six categories, as illustrated in Table 1.

### 2.5. Statistical Analysis

The data were analyzed by one-way analysis of variance (ANOVA) using the Costat software (version 6.311 copyright 1998-202008 Cohort Software. 798 Lighthouse Ave. PMB 320, Monterey, CA 93940, USA), and the comparisons of means were determined by Duncan’s multiple range tests at a 95% significance level (*p* ≤ 0.05).

## 3. Results

### 3.1. Penetration Study Experiment

**Figure 1:**
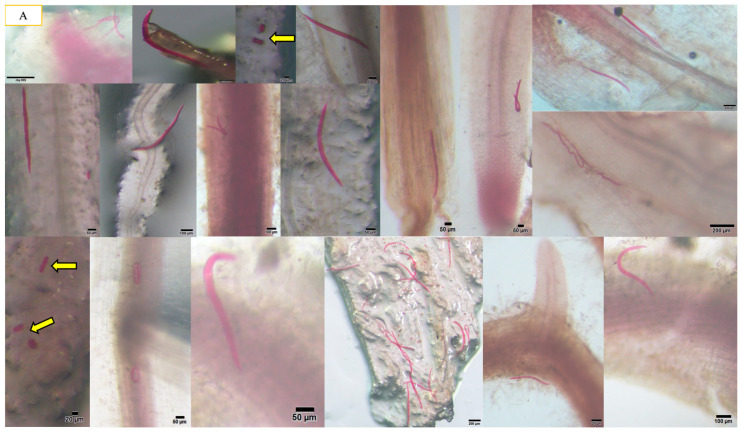

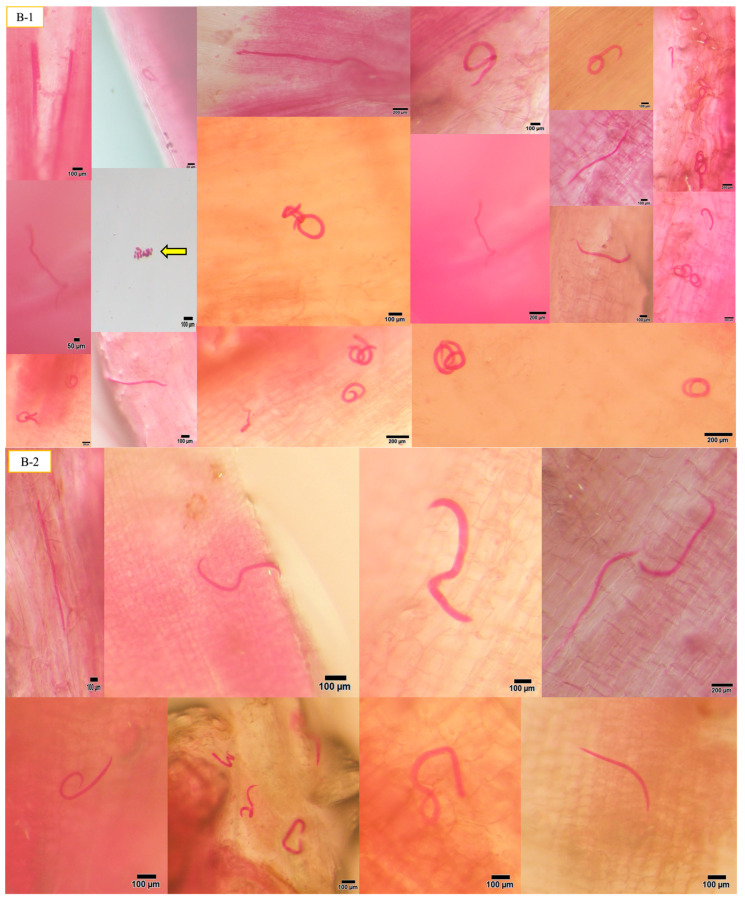

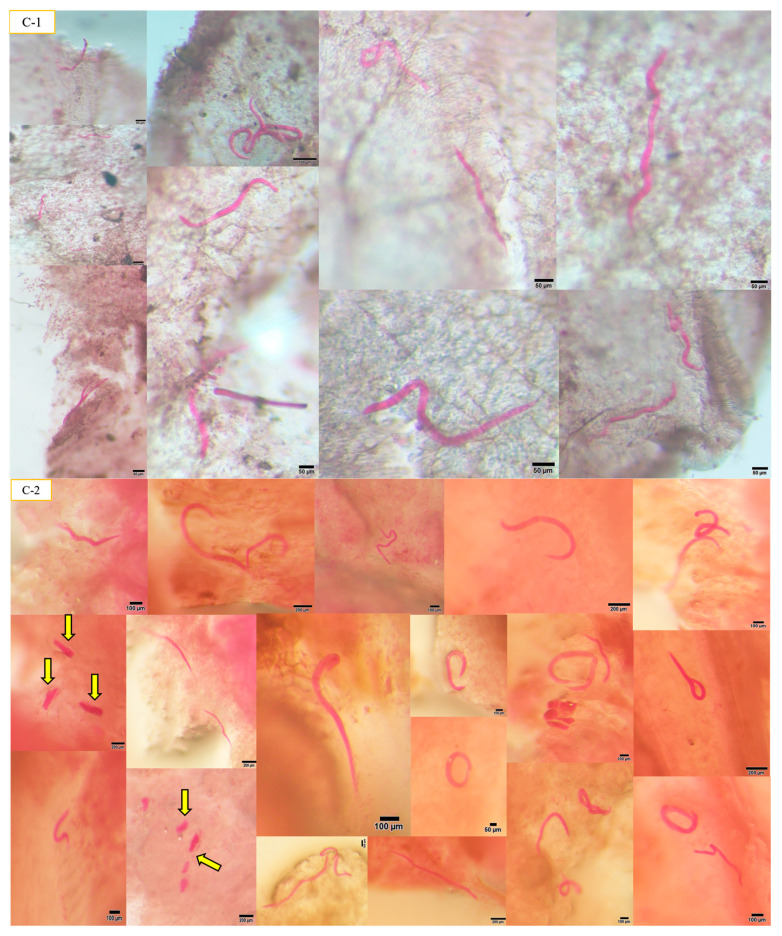
The presence of *A. besseyi* inside all soybean plant tissues. (**A**) *A. besseyi* in soybean root cortex and xylem vessels starting from the 1st dpi; (**B-1**,**B-2**) *A. besseyi* reached soybean stems starting from the 3rd dpi for all evaluated 17 soybean cultivars; (**C-1**,**C-2**) *A. besseyi* in soybean in foliar mesophyll and leaf veins starting from the 3rd dpi for all evaluated 17 soybean cultivars. Yellow arrows indicate the production and laying of *A. besseyi* eggs inside different soybean plant tissues (roots, stems, and leaves) starting from the 7th dpi.

**Figure 2:**
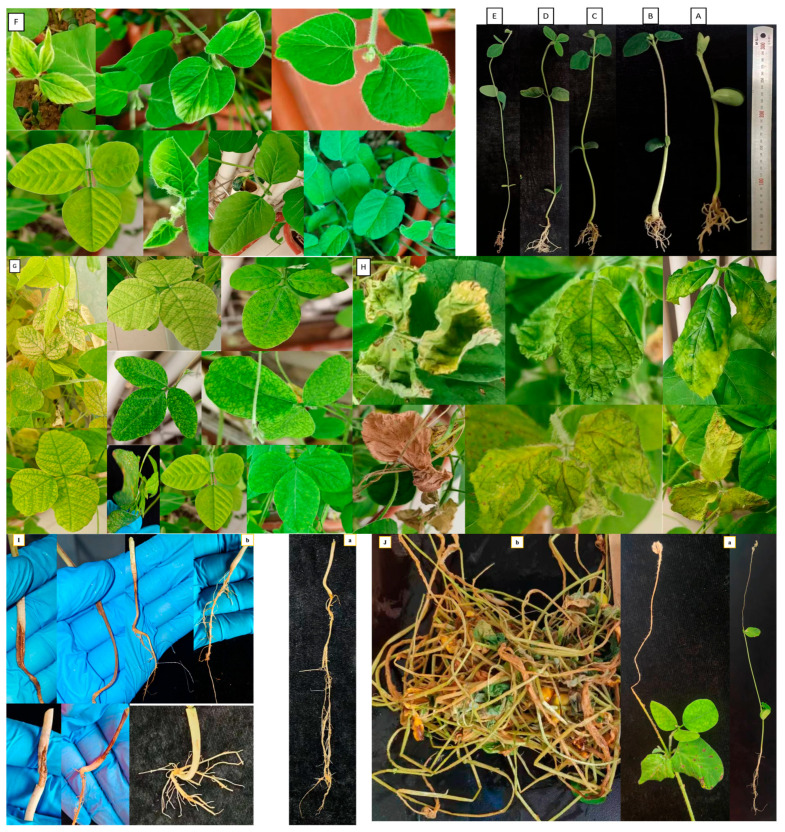
Typical GSFR syndrome symptoms in soybean plants inoculated with *A. besseyi* under controlled greenhouse conditions. (**A**–**E**) Soybean seedlings at 1, 4, 7, 10, and 13 dpis without any obvious GSFR syndrome symptoms; (**F**) starting from the 10th dpi, leaves for the majority of the 17th evaluated soybean cultivars remained green with obvious signs of discoloration, and in particular, the younger leaves of some cultivars exhibited severe deformation, distortion, and mosaic virus-like symptoms with obvious observation of node elongation; (**G**) typical leaf abnormalities start to appear around the 18th dpi, such as severe necrosis on buds and immature trifoliate leaves, vein thickening, and distortion; (**H**) severe GSFR syndrome symptoms on inoculated soybean leaves after the 60th dpi. (**I**) (**a**) non-infected control soybean root; (**b**) infected soybean roots showing splitting and lesions. (**J**) (**a**) soybean seedlings of some evaluated cultivars (Williams 82, Shendou 9, Liudou 99, Essex, and Zhonghuang 13) showed die-back tip symptoms resulting from *A. besseyi* feedings; (**b**) some evaluated soybean cultivars (Williams 82, Shendou 9, Liudou 99, and Zhonghuang 13) showed severe GSFRS symptoms, where the majority of the evaluated plants were completely destroyed.

**Figure 3:**
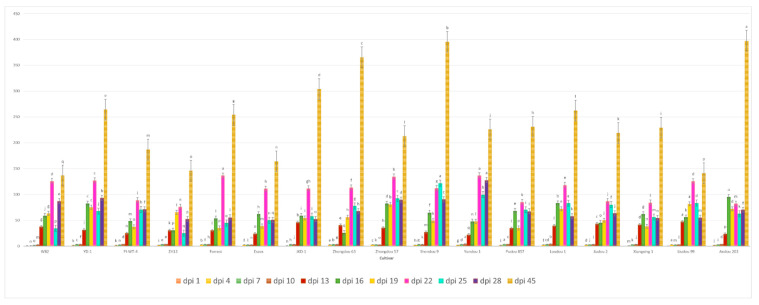
Average reproduction factor (RF) of *A. besseyi* population extracted from seventeen different soybean cultivars at eleven different evaluation times. W82: Williams 82, YD-1: Youdou 29, PI-WT-4: PI437654, ZH13: Zhonghuang 13, JKD-1: JKD 2. According to Duncan’s test (*p* < 0.05), different superscript letters indicate significant differences. The data are presented as the mean ± standard error.

We observed that *A. besseyi* effectively penetrates the roots of most evaluated soybean cultivars. This penetration was particularly pronounced in the Williams 82 cultivar, where the nematode demonstrated a high capacity to reach the xylem vessels within the first day following inoculation (Figure 1 and Figure 2). Additionally, we found significant differences among the seventeen evaluated soybean cultivars in their response to *A. besseyi* infection at one day post-inoculation. According to Figure 3, six soybean cultivars (Yudou 29, Zhonghuang 13, Essex, Shendou 9, Loudou 1, and Andou 203) prevented early *A. besseyi* root penetration. For the remaining soybean cultivars (PI437654, Forrest, JKD 2, Zhongdou 63, Zhongdou 57, Yundou 1, Pudou 857, Jiadou 2, Xiangxing 1, and Liudou 99), it was observed that *A. besseyi* successfully penetrated their roots, but there were significant differences in the final nematode populations extracted from their roots. Some cultivars, such as Jiadou 2, Zhongdou 57, and Xiangxing 1, allowed nematode entry but limited subsequent development and reproduction. Starting from the 3rd dpi, we detected *A. besseyi* inside the roots, xylem vessels, nodes, and foliar mesophyll of all seventeen evaluated soybean cultivars at all evaluation times. At the 7th dpi, *A. besseyi* began producing eggs in the root and shoot systems (Figure 1). Furthermore, we confirmed a significant increase in the total *A. besseyi* population extracted from different soybean tissues at each evaluation point. According to Figure 3, the lowest reproduction values were observed in Zhonghuang 13, Essex, and PI437654. Moreover, we noticed that although the Shendou 9 cultivar exhibited resistance to *A. besseyi* penetration from the 1st dpi, it had the highest reproduction values compared to other cultivars. This could be due to the lack or absence of resistance genes or secondary anti-nematode phytochemical metabolites (e.g., phenolics, alkaloids, terpenoids, saponins). Regarding soybean plant growth, non-inoculated control soybean plants showed an elevation in various vegetative growth characteristics, such as PH, FRW, and FSW (Table S2). For the observed syndrome symptoms, we found that leaves for the majority of the seventeen evaluated soybean cultivars remained green with obvious signs of discoloration, particularly in the younger leaves of some cultivars, which exhibited severe deformation, distortion, and mosaic virus-like symptoms with clear node elongation starting from the 10th dpi (Figure 2). Typical leaf abnormalities such as severe necrosis on buds and immature trifoliate leaves began to appear around the 18th dpi. Starting from the 22nd dpi, affected soybean plants exhibited distorted stems with enlarged nodes and twisted leaves, and the whole plant became more stunted compared to the non-inoculated soybean plants.

**Figure 4:**
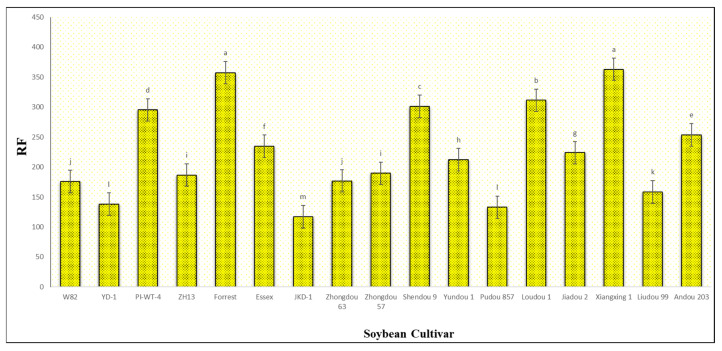
Average reproduction factor (RF) of *A. besseyi* population extracted from seventeen different soybean cultivars after the 60th dpi under controlled greenhouse conditions. W82: Williams 82, YD-1: Youdou 29, PI-WT-4: PI437654, ZH13: Zhonghuang 13, JKD-1: JKD 2. According to Duncan’s test (*p* < 0.05), different superscript letters indicate significant differences. The data are presented as the mean ± standard error.

After the 60th dpi, we found that there were significant differences in the weights of the fresh roots and shoots of the infected and non-infected soybean plants, with significant elevation in the nematode final populations extracted from each cultivar (Table S2; Figure 4). During this period, *A. besseyi*-infected soybean plants showed severe GSFR syndrome symptoms. Moreover, splitting and lesions were observed in the cortical tissue of the infected plants’ roots. In comparison to the non-infected soybean plants, the axillary buds exhibited necrosis and reduced elongation.

### 3.2. Nematode Transmission by Soybean Seeds

**Figure 5:**
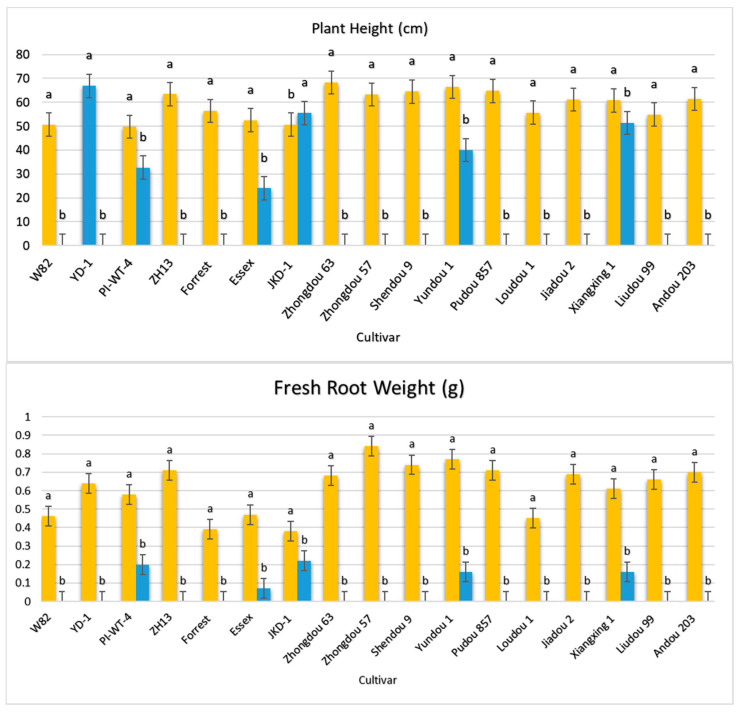

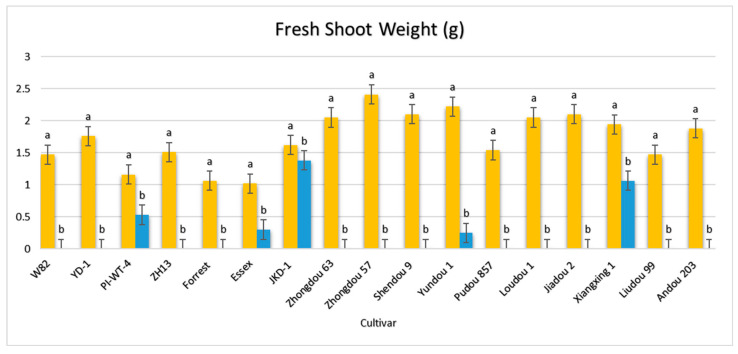
Comparison between the vegetative growth parameters of the 17 different non-inoculated and inoculated soybean seeds under controlled greenhouse conditions. Orange bars: control non-inoculated soybean seeds; blue bars: inoculated soybean seeds. According to Duncan’s test (*p* < 0.05), different superscript letters indicate significant differences. The data are presented as the mean ± standard error.

The results obtained from screening 17 different soybean cultivars for their ability to transmit *A. besseyi* via their seeds revealed significant findings. Twelve soybean cultivars failed to emerge from the soil, a reduction in emergence rate directly attributed to nematode parasitism, with a 100% seed infection rate for each cultivar (Figure 5). Additionally, only five cultivars (PI437654, Essex, JKD 2, Yundou 1, and Xiangxing1) successfully emerged from the soil, exhibiting germination rates of 87.5, 62.5, 25, 12.5, and 100%, respectively. All emerged seeds from these five cultivars also showed a 100% infection rate. This reduced emergence was due to the high *A. besseyi* infection of the inoculated soybean seeds. Furthermore, the five emerging cultivars displayed extremely severe GSFR syndrome symptoms. Notably, only two seedlings from the JKD 2 cultivar and one seedling from the Yundou 1 cultivar emerged out of the total eight replicates employed for each cultivar. All these observations confirm that soybean seeds play a serious and significant role as a source of *A. besseyi* inoculum, highlighting the critical need to explore strategies for *A. besseyi* elimination from soybean seeds.

### 3.3. Field Experiment

**Figure 6:**
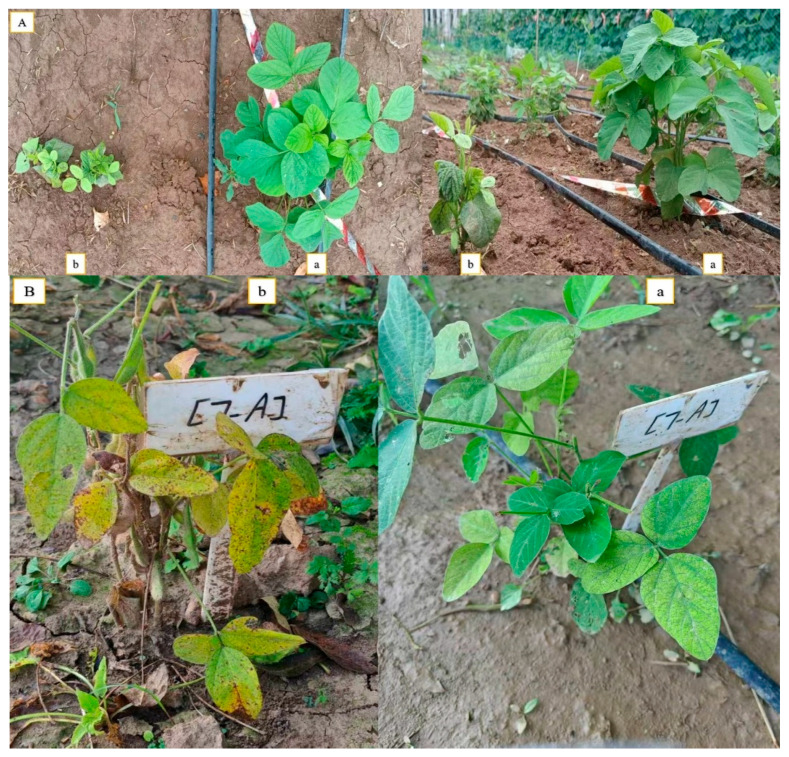

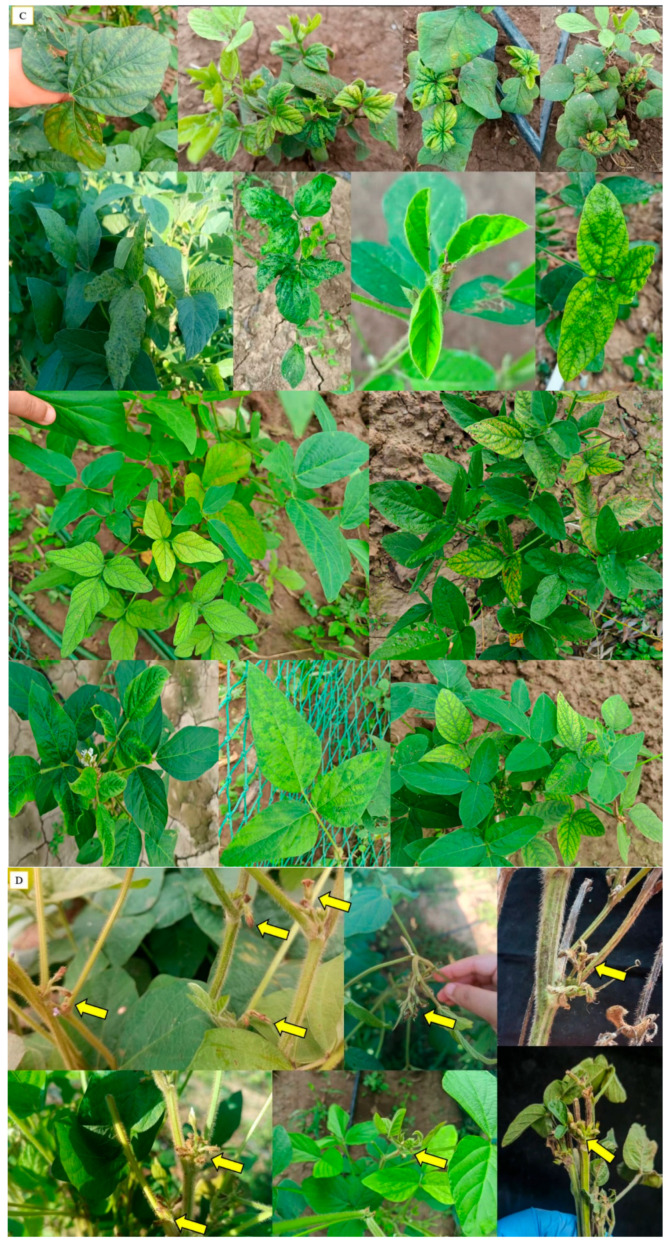

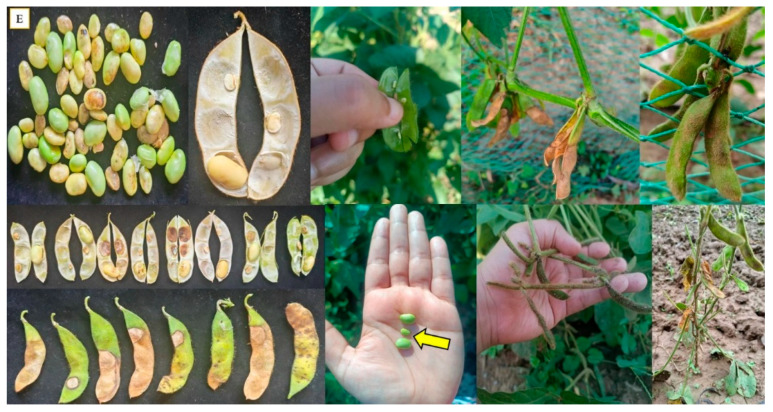
Typical symptoms of the new GSFR syndrome associated with the infection of *A. besseyi* in seventeen different soybean cultivars in a naturally infested field. (**A**) Comparison of final appearance between non-inoculated and inoculated soybean plants: (**a**) non-inoculated control soybean plant, (**b**) *A. besseyi*-inoculated soybean plant; (**B**) disease severity degree on JKD 2 cultivar inoculated with *A. besseyi* two weeks after germination: (**a**) GSFR symptoms one month after inoculation, (**b**) GSFR symptoms two months after inoculation; (**C**) trifoliate soybean leaves showing virus-like symptoms including mosaic, distortion, vein thickening, necrosis, deformation, blistering, and strapping; (**D**) enlargement and necrosis of inflorescence nodes and buds with significant level of flower abortion; (**E**) symptoms on soybean pods, including distortion, thickening, and brown corky necrosis with significantly low level of pod production. The yellow arrow indicates a soybean seed produced from an infected soybean cultivar compared to the non-inoculated control.

**Figure 7:**
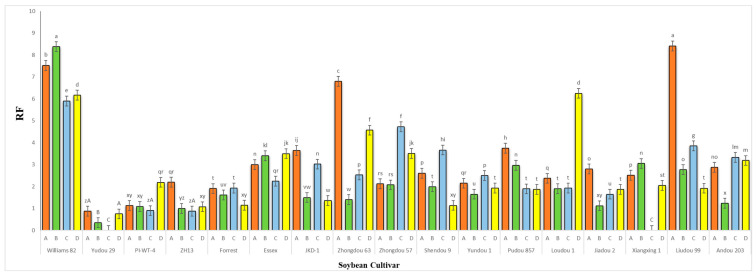
Average reproduction factor (RF) of *A. besseyi* population extracted from seventeen different soybean cultivars from naturally infested field conditions; (**A**–**D**) four different inoculation periods: 2 weeks after emergence, 3 weeks after emergence, V5-V6 vegetative stage, and V6-V7 vegetative stage, respectively. W82: Williams 82, YD-1: Youdou 29, PI-WT-4: PI437654, ZH13: Zhonghuang 13, JKD-1: JKD 2. According to Duncan’s test (*p* < 0.05), different superscript letters indicate significant differences. The data are presented as the mean ± standard error.

**Figure 8:**
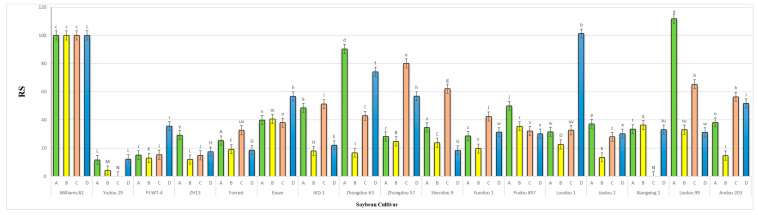
Relative susceptibility values (RS) of the seventeen different soybean cultivars infested by *A. besseyi* during four different inoculation periods under natural field conditions. (**A**–**D**) Four different inoculation periods: 2 weeks after emergence, 3 weeks after emergence, V5-V6 vegetative stage, and V6-V7 vegetative stage, respectively. W82: Williams 82, YD-1: Youdou 29, PI-WT-4: PI437654, ZH13: Zhonghuang 13, JKD-1: JKD 2. According to Duncan’s test (*p* < 0.05), different superscript letters indicate significant differences. The data are presented as the mean ± standard error.

**Figure 9:**
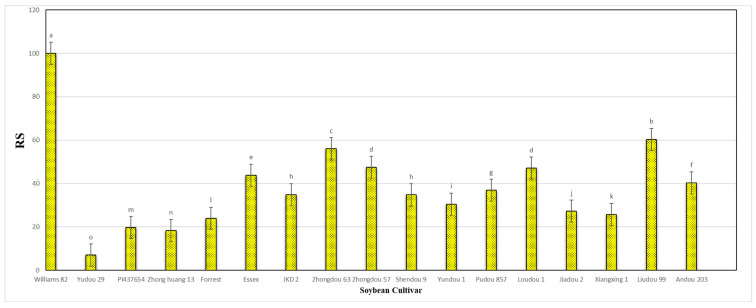
Final mean score of the relative susceptibility (RS) values for each of the seventeen different soybean cultivars infested by *A. besseyi* under natural field conditions. According to Duncan’s test (*p* < 0.05), different superscript letters indicate significant differences. The data are presented as the mean ± standard error.

**Figure 10:**
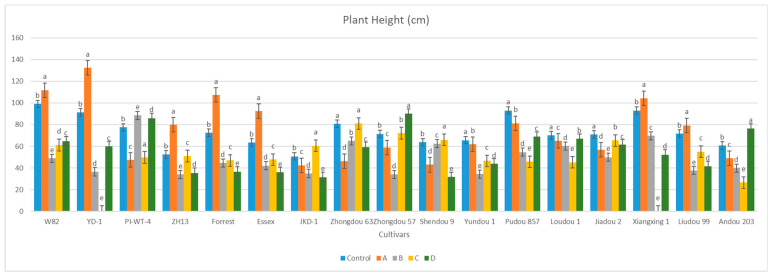

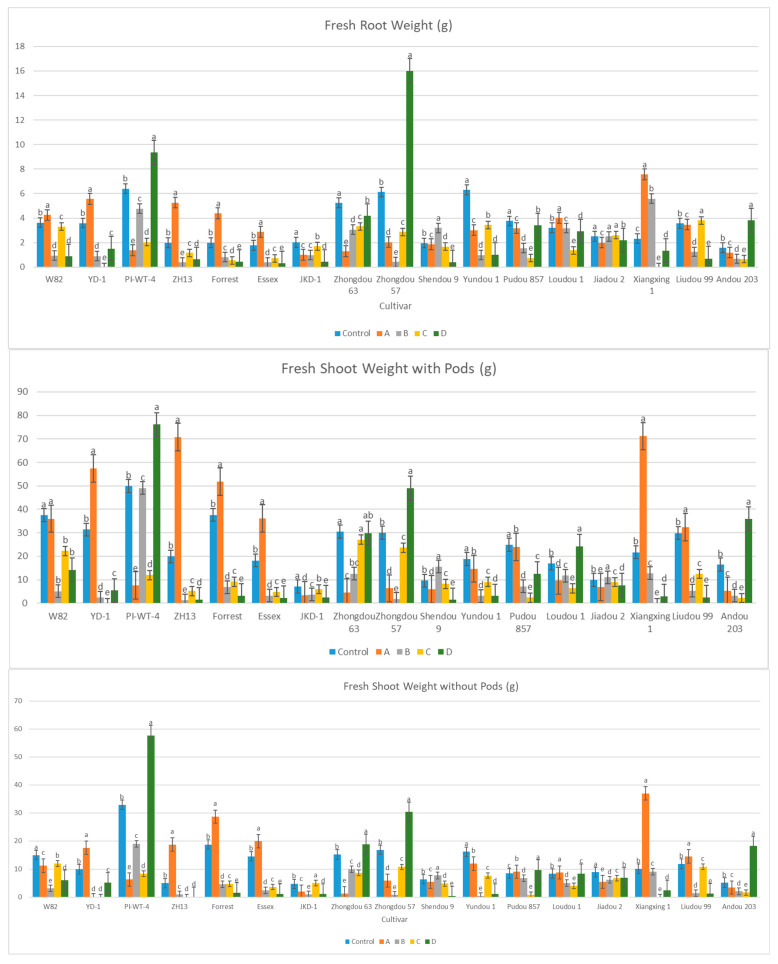

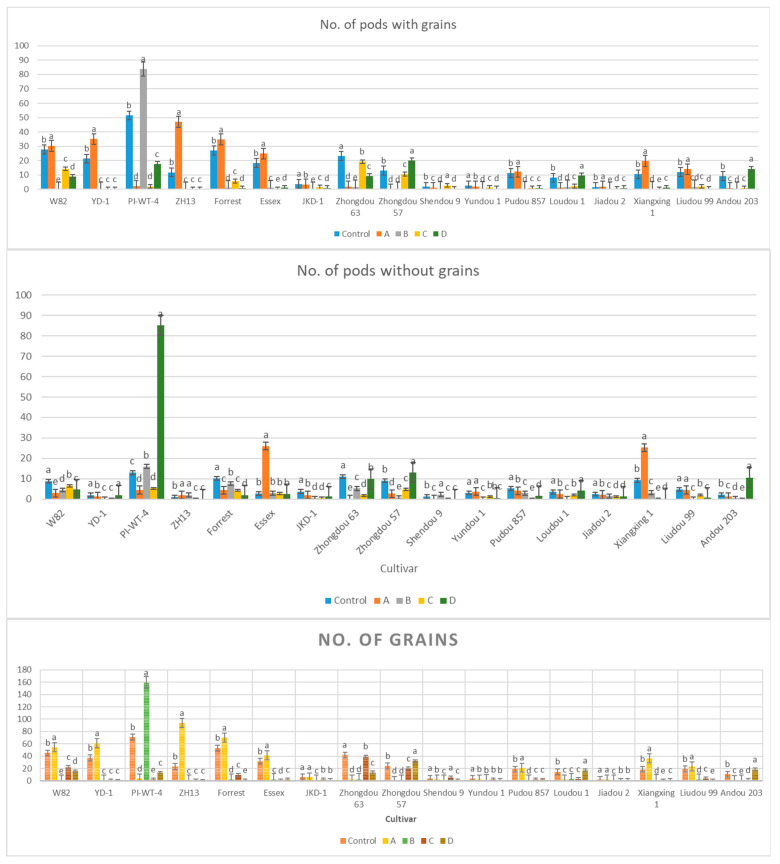
Comparison between the vegetative growth parameters of the 17 different non-inoculated and inoculated soybean cultivars under natural field conditions. (**A**–**D**) Four different inoculation periods: 2 weeks after emergence, 3 weeks after emergence, V5-V6 vegetative stage, and V6-V7 vegetative stage, respectively. According to Duncan’s test (*p* < 0.05), different superscript letters indicate significant differences. The data are presented as the mean ± standard error.

**Figure 11:**
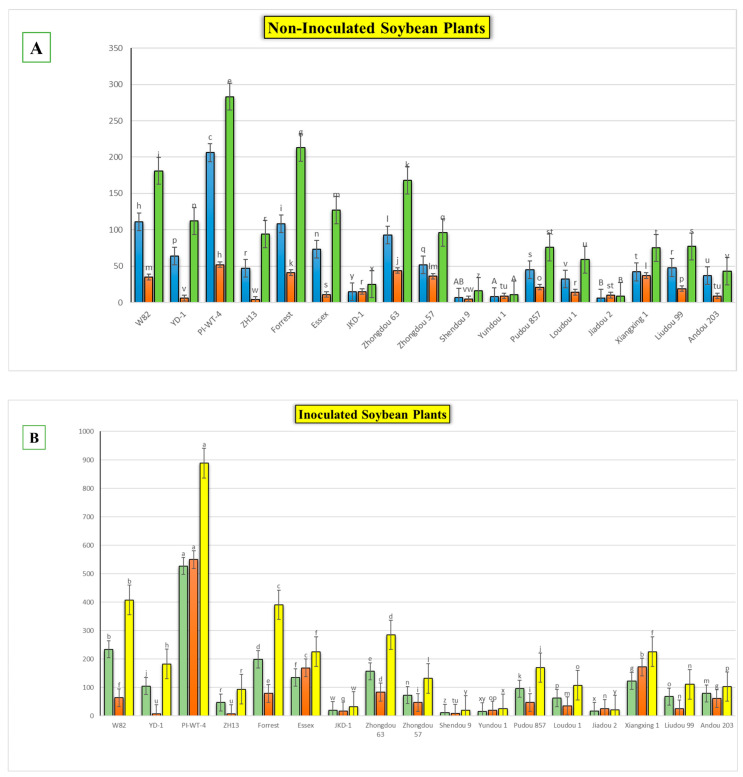
Comparison between the total pod and seed production levels of each evaluated cultivar during the whole field experimental period. (**A**) Data obtained from the non-inoculated soybean cultivars, with the values representing 5 replicates for each cultivar, (**B**) data obtained from the inoculated soybean cultivars, with the values representing 20 replicates for each cultivar (4 different inoculation periods; each period had 5 replicates). W82: Williams 82, YD-1: Youdou 29, PI-WT-4: PI437654, ZH13: Zhonghuang 13, JKD-1: JKD 2. Each measured factor was statistically analyzed separately. According to Duncan’s test (*p* < 0.05), different superscript letters indicate significant differences. The data are presented as the mean ± standard error.

The results obtained from a natural field experiment revealed that all 17 inoculated soybean cultivars exhibited severe symptoms of green stem and foliar retention (GSFR) syndrome, including virus-like symptoms (mosaic, vein thickening, and distortion) on affected soybean leaves. We observed a high frequency of flower abortion and a significant reduction in pod production. This reduction was maybe due to *A. besseyi* parasitism on infested soybean nodes, inflorescences, and meristematic tissues. The remaining pods were deformed and necrotic and exhibited poor growth with a high production of pods without seeds (Figure 6). We then extracted *A. besseyi* from all infested symptomatic soybean shoot tissues. Based on the average reproduction factor (RF) values (Figure 7), the final nematode population extracted from each trial varied significantly according to the plant tissue. By comparing the total nematode final populations extracted from each cultivar throughout the field experiment and their average RF values, we found that cultivar Yudou 29 had the lowest RF values, followed by Zhonghuang 13, PI437654, and Forrest, respectively. This indicates that Yudou 29 is the most resistant cultivar to *A. besseyi* infestation under field conditions among all the 17 evaluated soybean cultivars, followed by Zhonghuang 13, PI437654, and Forrest. Conversely, the Williams 82 cultivar had the highest RF values, followed by Liudou 99, Zhongdou 63, and Loudou 1. This indicates that Williams 82 cultivar is the most susceptible cultivar and significantly susceptible to *A. besseyi* infestation under infested field conditions among all the evaluated soybean cultivars, followed by cultivars Liudou 99, Zhongdou 63, and Loudou 1. Secondly, according to the relative susceptibility (RS) scores (Figure 8 and Figure 9), we differentiated the evaluated soybean cultivars based on their resistance response to *A. besseyi* infestation into the following categories: highly resistant: Yudou 29, resistant: PI437654 and Zhonghuang 13, intermediate resistant: Forrest, Jiadou 2, and Xiangxing 1, and highly susceptible: Williams 82, Liudou 99, and Zhongdou 63. For differentiation between the evaluated soybean cultivars according to their susceptibility toward the *A. besseyi* infestation, we found that Williams 82, Liudou 99, and Zhongdou 63 were the cultivars most susceptible to *A. besseyi* infection compared to the other evaluated cultivars. Significantly, the results for the average reproduction factor were consistent with those for the relative susceptibility index, confirming their suitability and applicability for differentiating soybean cultivars for resistance screening under *A. besseyi* infestation. Moreover, we noticed significant differences in soybean vegetative parameters among the four different inoculation periods (2 weeks and 3 weeks after emergence, V5–V6 and V6–V7 vegetative stages). Symptoms were more severe on soybean plants inoculated with *A. besseyi* at earlier stages (Figure 10). Interestingly, we also extracted and identified *A. besseyi* from non-inoculated control soybean plants, which were infested naturally by the *A. besseyi* population that existed in the soil. Additionally, we calculated the total pod and seed production for each non-inoculated and inoculated soybean cultivar to assess the nematode’s impact on total yield quantity and quality. According to Figure 11, PI437654 had the maximum pod and seed production levels in both non-inoculated and inoculated soybean cultivars, followed by Forrest and Williams 82. Conversely, Jiadou 2, followed by Yundou 1 and Shendou 9, exhibited the lowest pod and seed production levels. Although PI437654 had the highest level of pod production, it also produced the maximum number of empty pods without seeds among all the evaluated cultivars, followed by Zhongdou 63 and Forrest in the non-inoculated group and Xiangxing 1 and Essex in the inoculated soybean group. In conclusion, the symptoms of GSFR syndrome observed in this investigation highlight its significance for Chinese soybean production and emphasize the urgent need for more efficient control techniques for this destructive nematode.

## 4. Discussion

*Aphelenchoides besseyi* is considered a destructive foliar parasitic nematode that causes severe damage to a wide range of important crops worldwide. *A. besseyi* is characterized by its remarkable ability to persist in anhydrobiotic conditions in crop debris remaining in the soil. It can then migrate to the upper parts of host plants via rain splashes or the water layer covering foliar tissue surfaces, penetrating plant tissues via natural openings like stomata [24,25]. In contrast to the previous literature, in the current study, we conducted the *A. besseyi* artificial inoculation via a small hole in the soil located near the root system. We then observed the developed symptoms in inoculated soybean plants, as reported by [10] for soybean. In order to ascertain whether nematodes invaded plant shoot systems through splashing or root penetration, we used fuchsin acid to stain the root and shoot systems. We successfully detected *A. besseyi* within all soybean plant tissues (root, stem, and leaves), confirming its endoparasitic habit and significant ability to penetrate roots to reach the upper sections of the foliar system, where it causes GSFR syndrome symptoms. Our findings were consistent with those discovered by the authors of [15], who suggested an endoparasitic relationship between *A. besseyi* and soybean plants. They also successfully detected *A. besseyi* in the cortex and xylem of root tissues and internally in plant stems. Another recent study on common bean specifically confirmed *A. besseyi*’s ability to penetrate roots (e.g., through the cortex) as early as one day after inoculation [9]. This study found that the number of nematodes recovered inside the plants was significantly higher than that on the plant surface, reinforcing the nematode’s endoparasitic nature and successful internal colonization. This suggests that while roots may not always be the preferred feeding site, they are a crucial pathway for systemic infection [9]. Furthermore, *A. besseyi* secretes various effector proteins and enzymes from its esophageal glands, which play a role in softening cell walls, suppressing plant defenses, and facilitating movement through tissues. For example, a study on *A. besseyi* identified genes related to carbohydrate-active enzymes such as glycoside hydrolases (GHs) and carbohydrate esterases (CEs), including a GH45 cellulase gene. While not directly linked to root penetration, these enzymes could aid in degrading plant cell wall components, facilitating entry and movement [18]. We also effectively documented the nematode’s ability to penetrate soybean roots at just the 1st dpi and its subsequent transmission to the upper portions of the foliage, where it was detected starting from the 4th dpi. Regarding the final nematode population extracted from different soybean plant tissues, the increased population density within the roots, stems, and leaves of evaluated soybean cultivars was a significant indication of *A. besseyi* ascendent movement within plant tissues. These findings indicate a continuous flow of nematode migration from root tissues to the upper foliar tissues during the soybean growth phase, resulting in significant *A. besseyi* accumulation in the leaves. It is crucial to delve into the potential mechanisms of resistance against *A. besseyi*, especially concerning root penetration. While much of the visible damage from *A. besseyi* manifests in the foliage, its ability to penetrate roots and then migrate systemically means that root-level defenses are crucial. Since *A. besseyi* is a relatively new problem in crops like soybean and common bean (where root penetration has been more definitively observed), detailed studies on root-specific resistance mechanisms are still emerging. Plants employ a vast array of secondary metabolites as chemical defenses, broadly categorized into pre-formed (constitutive) and inducible defenses. For root penetration by *A. besseyi*, these could act at various stages: preventing the nematode from reaching or attempting to penetrate the root, directly harming or inhibiting the nematode during or after penetration, or disrupting essential nematode processes. Additionally, root structural barriers (physical features within plant roots) act as a first line of defense or impede the movement and establishment of invading nematodes. These barriers can be constitutive (always present) or inducible (formed in response to attack).

Our evaluation of four inoculation periods to determine the favorable infection stage for the current nematode was based on previous studies stating that GSFR syndrome symptoms become more noticeable starting from the early stages of plant reproduction [10]. However, in our current study, the syndrome symptoms were detected earlier, indicating that the temperature and moisture conditions during the whole experimental period were well-suited and favorable to facilitating nematode infection and the emergence of early severe symptoms in evaluated soybean cultivars. The presence of *A. besseyi* significantly impacted soybean plant growth, and the chronology of symptoms may explain the observed abnormalities in vegetative parameters. Moreover, compared to negative control soybean plants, inoculated plants displayed necrosis on their axillary buds and an obvious reduction in plant elongation starting from the 15th dpi. Furthermore, we observed an increase in nematode final population within different soybean plant tissues, which negatively impacted soybean growth and development, as evidenced by differences in FRW between treated and non-treated soybean cultivars along with reduced rate of growth in FSW. Based on these various vegetative parameters (PH, FRW, and FSW), we conclude that *A. besseyi* infection has a significant negative impact on soybean growth and development. Therefore, we can declare a direct correlation between the manifestation, progression, and severity of GSFR syndrome symptoms and the existence of high *A. besseyi* populations within soybean plant tissues.

In the comparison between infected and non-infected soybean cultivars, non-infected control plants grew normally until reaching maturity. In contrast, for infected plants, among some evaluated soybean cultivars that maintained green foliage, there were severe mosaic-like symptoms that appeared on some leaves with larger size along with dwarfed growth with splitting in roots, resulting in an elevation of FRW and FSW values compared to control soybean plants. Taking this into account, we advise against using vegetative growth parameters alone as a significant indicator for GSFR syndrome characterization in soybean without also accurately observing syndrome symptoms. Interestingly, our findings were consistent with those declared by [15]. Additionally, *A. besseyi* is a seed-transmitted nematode with an exceptional ability to persist in anhydrobiotic conditions on stored rice grain for a prolonged period, which is considered a substantial factor in its widespread dissemination [16,18]. However, the resistance observed in PI437654 likely refers to its ability to endure the nematode’s presence with a reduced overall negative impact on plant vigor; this resistance does not necessarily guarantee optimal reproductive efficiency. The presence of empty pods suggests that while the plant copes with the nematodes, it still experiences a resource or physiological bottleneck that prevents complete seed development. This bottleneck could be a direct, but subtle, consequence of living with nematodes (e.g., energy drain for defense), or it could be due to the interplay of nematode stress with other environmental factors, leading to a complex stress load that impacts reproductive partitioning and, ultimately, pod fill. This scenario highlights that resistance is not always absolute and that plant responses to stress are multifaceted, involving intricate trade-offs in resource allocation and physiological processes.

The process of developing a resistant cultivar presents plenty of obstacles for genetic breeding operations. Accurate genotype phenotyping is considered one of the major challenges, particularly for plant-parasitic nematodes, since the methods used in experiments can significantly impact this process [26]. Numerous studies involving migratory nematodes have illustrated that the absence of consistency in phenotyping approaches can have a significant impact, leading to the misclassification of evaluated cultivars. Ref. [27] highlighted the significance of assessing symptoms and crop production when evaluating the genotype resistance along with the pathogenicity of these nematodes, rather than relying solely on their reproductive ability. In the current study, we designed a diagrammatic scale that proved to be highly suitable and applicable for differentiating between various soybean cultivars under *A. besseyi* infestation for resistance screening purposes. The symptoms described in the present investigation highlight the significance of the GSFR syndrome for soybean production and boost the urgent need for effective control technologies of this destructive nematode. Despite offering valuable insights into potential resistance mechanisms, our current understanding of *A. besseyi* infestation, particularly in newly recognized host crops like soybean and common bean, is constrained by several factors. Existing research frequently utilizes a limited number of plants per cultivar, which may not adequately represent the full spectrum of genetic variation or the adaptable nature of resistance traits across a wider range of genotypes. Moreover, the influence of environmental factors—such as variations in soil moisture, temperature, and nutrient availability—can profoundly impact both nematode activity and the plant’s defensive reactions. These unaddressed variables might either obscure or exaggerate the observed resistance characteristics. To advance this field, future investigations should focus on large-scale evaluations of diverse plant genetic resources conducted under precisely controlled environmental conditions to pinpoint cultivars exhibiting consistent resistance. Following this, sophisticated molecular approaches, such as transcriptomic profiling, could be implemented to compare gene expression patterns between resistant and susceptible cultivars. This detailed analysis promises to reveal the specific molecular pathways that underpin resistance to *A. besseyi* penetration, encompassing the biosynthesis of root defense chemicals, modifications to cell wall structures, and the intricacies of signal transduction.

## Figures and Tables

**Table 1:** Relative susceptibility index scale (RS) for differentiating between soybean cultivars according to their response toward infestation by *A. besseyi* nematodes.

Relative Susceptibility (RS) Index	Score
Highly Resistant (HR)	<10
Resistant (R)	10–20
Intermediate Resistant (IR)	20–30
Intermediate Susceptible (IS)	30–40
Susceptible (S)	40–50
Highly Susceptible (HS)	>50

Note: Williams 82 was used as the standard susceptible cultivar for RS calculations.

## Data Availability

All data supporting the findings of this study are available within the paper and its Supplementary Materials file.

## Supplementary Materials

The following supporting information can be downloaded at https://www.mdpi.com/article/10.3390/life15071154/s1: Table S1: Seventeen different soybean cultivars used in the current study for evaluating the pathogenicity of *A. besseyi* under field and greenhouse conditions; Table S2. Comparison between the vegetative growth parameters of the seventeen different non-inoculated and inoculated soybean cultivars under controlled greenhouse conditions one day after artificial inoculation.
