# Supplementary material for: Studies on Host–Parasite Relationship Between Soybean Plants and Aphelenchoides besseyi
Source: Life (Basel). 2025 Jul 21;15(7):1154. doi: 10.3390/life15071154 (PMC12300678; doi:10.3390/life15071154)
Supplement: Supplementary file 1 [file life-15-01154-s001.zip › life-3729767-supplementary.pdf]

Supplementary Data

Table S1. Seventeen different soybean cultivars used in the current study for evaluation the pathogenicity of *A. besseyi* under field and greenhouse conditions.

**Code**	**Commercial name**	**Origin**
1	Williams 82	
2	Yudou 29	
3	PI 437654	
4	Zhonghuang 13	
5	Forrest	
6	Essex	
7	JKD 2	
8	Zhongdou 63	
9	Zhongdou 57	Anhui Province
10	Shendou 9	
11	Yundou 1	
12	Pudou 857	
13	Loudou 1	
14	Jiadou 2	
15	Xiangxing 1	
16	Liudou 99	
17	Andou 203	

Table S2. Comparative between the vegetative growth parameters of the seventeen different non-inoculated and inoculated soybean cultivars under controlled greenhouse conditions after one day post artificial inoculation.

Soybean Cultivar	Non-Inoculated Plants			Inoculated Plants		
	PH	FRW	FSW	PH	FRW	FSW
Williams 82	19.67±0.33	0.33±0.05	0.8±0.02	21.33±0.88	0.3±0.05	0.95±0.11
Yudou 29	23±1.15	0.39±0.02	0.97±0.06	26.33±1.33	0.33±0.03	1.03±0.11
PI437654	20.33±0.33	0.47±0.07	0.82±0.03	23±1.15	0.31±0.04	0.74±0.11
Zhonghuang 13	20.67±1.45	0.22±0.02	0.64±0.04	20.33±0.33	0.24±0	0.91±0.16
Forrest	24±1	0.28±0.02	0.78±0.07	17.33±1.2	0.19±0.04	0.74±0.08
Essex	20.33±0.33	0.43±0.1	0.7±0.02	20.67±0.67	0.35±0.01	0.64±0.04
JKD 2	22.67±1.45	0.32±0.01	1.03±0.1	21±0	0.19±0.03	0.74±0.14

Zhongdou 63	23±1	0.42±0.03	1.14±0.03	22.67±0.33	0.33±0.05	1.18±0.07
Zhongdou 57	23±1.15	0.4±0.03	1.37±0.1	22.33±1.33	0.31±0.03	1.27±0.05
Shendou 9	23±1	0.3±0.02	1.31±0.06	19±0.58	0.26±0.01	1.16±0.04
Yundou 1	19±1	0.32±0.03	1.24±0.11	20±0	0.29±0.02	1.18±0
Pudou 857	22.33±1.45	0.36±0.12	1.14±0.05	21.67±0.67	0.25±0.02	0.9±0.06
Loudou 1	22.33±1.45	0.41±0.01	1.07±0.03	19.33±0.67	0.22±0.01	0.84±0.02
Jiadou 2	19.67±0.33	0.34±0.08	1.01±0.11	20±0.58	0.34±0.04	1.03±0.08
Xiangxing 1	21±1	0.5±0.07	1.08±0.01	22.67±1.2	0.34±0.03	1.21±0.12
Liudou 99	21.33±0.33	0.37±0.03	0.92±0.08	21.33±0.33	0.42±0.03	1.04±0.03
Andou 203	21±0	0.39±0.03	1.16±0.06	20.67±0.67	0.27±0.07	1.19±0.08

Note: PH: Plant height; FRW: fresh root weight; FSW: fresh shoot weight; Values are the means of three replicates ± SE.

Table S2-1. (Continued at 4th dpi).

Soybean Cultivar	Non-Inoculated Plants			Inoculated Plants		
	PH	FRW	FSW	PH	FRW	FSW
Williams 82	20.25±1.84	0.38±0.08	0.82±0.04	21.25±0.75	0.21±0.06	1.16±0.25
Yudou 29	18.67±0.33	0.41±0.12	0.92±0.04	21±1	0.4±0.15	1±0.03
PI437654	27±2	0.31±0.06	0.66±0.05	29.75±0.75	0.29±0.13	0.73±0.03
Zhonghuang 13	19±2.08	0.46±0.18	0.85±0.13	25±0	0.68±0.16	1.15±0.02
Forrest	16.5±1.19	0.56±0.22	0.55±0.04	20.33±0.33	0.26±0.07	0.77±0.07
Essex	19.75±2.29	0.34±0.1	0.73±0.11	17.5±2.5	0.26±0.12	0.67±0.13
JKD 2	18±1.53	0.23±0.15	0.73±0.13	17±2	0.23±0.11	0.81±0.1
Zhongdou 63	16.5±1.84	0.45±0.13	1.13±0.04	16±2.65	0.45±0.13	0.96±0.07
Zhongdou 57	17.83±1.48	0.26±0.09	1.15±0.15	15.5±2.18	0.38±0.11	1.24±0.18
Shendou 9	18.17±1.36	0.7±0.35	1.04±0.06	21.75±3.25	0.38±0.28	1.37±0.29
Yundou 1	16±1.34	0.32±0.08	1.06±0.09	20.33±0.88	0.55±0.18	1.41±0.07
Pudou 857	21.13±1.9	0.41±0.05	0.95±0.05	25±0	0.41±0.16	1.01±0.17
Loudou 1	21.67±1.76	0.39±0.09	1.04±0.11	20±0.58	0.28±0.04	1.09±0.04
Jiadou 2	14±1	0.48±0.31	1.08±0.06	18±2.31	0.49±0.16	1.01±0.07
Xiangxing 1	25.33±2.33	0.55±0.13	0.94±0.3	24±1	0.39±0.32	1.02±0.03
Liudou 99	22.33±1.76	0.58±0.26	1.11±0.16	18±1	0.1±0.05	0.5±0.01
Andou 203	20.5±0	0.15±0	1.12±0	20.33±0.83	0.6±0.22	1.35±0.13

Table S2-2. (Continued at 7th dpi).

Soybean Cultivar	Non-Inoculated Plants			Inoculated Plants		
	PH	FRW	FSW	PH	FRW	FSW
Williams 82	27.75±1.03	0.29±0.09	0.73±0.05	29.75±0.25	0.19±0.08	0.52±0.19
Yudou 29	34±1.96	0.42±0.1	0.9±0.06	22.33±1.86	0.27±0.11	0.43±0.11
PI437654	32.33±1.45	0.49±0.33	0.69±0.03	26.67±3.84	0.2±0.07	0.36±0.02
Zhonghuang 13	29.67±0.33	0.47±0.17	0.85±0.15	27.5±2.5	0.47±0.34	1.05±0.06

Forrest	19.5±2.18	0.21±0.05	0.48±0.04	25.33±2.91	0.11±0.04	0.42±0.11
Essex	27.5±1.44	0.34±0.1	0.34±0.03	20.17±0.17	0.19±0.11	0.12±0.02
JKD 2	31.67±1.67	0.21±0.08	0.58±0.08	28.5±1.5	0.31±0.08	0.33±0.03
Zhongdou 63	27.67±1.2	0.48±0.2	0.64±0.04	15.67±2.33	0.23±0.06	0.36±0.03
Zhongdou 57	26.83±1.01	0.19±0.07	0.88±0.07	29.67±0.33	0.29±0.07	0.62±0.04
Shendou 9	22±0.71	0.35±0.1	0.61±0.06	18.67±0.88	0.33±0.09	0.64±0.16
Yundou 1	25.17±2.46	0.33±0.1	0.68±0.09	28.5±3.5	0.35±0.02	0.81±0.05
Pudou 857	27.25±1.6	0.39±0.07	0.75±0.02	34.5±4.5	0.3±0.05	0.69±0.05
Loudou 1	28±3	0.77±0.11	1.04±0.39	24.83±2.89	0.22±0.06	0.5±0.09
Jiadou 2	36±3	0.3±0.03	1.4±0.39	26.5±3	0.53±0.42	1.3±0.04
Xiangxing 1	34.33±2.03	0.31±0.09	0.79±0.08	32.67±1.45	0.32±0.06	0.94±0.06
Liudou 99	35.5±3.5	0.11±0.05	1.29±0.02	16.33±1.33	0.35±0.11	0.69±0.1
Andou 203	33.33±2.03	0.26±0.09	1.24±0.16	37.5±0.5	0.38±0.25	1.4±0.2

Table S2-3. (Continued at 10th dpi).

Soybean Cultivar	Non-Inoculated Plants			Inoculated Plants		
	PH	FRW	FSW	PH	FRW	FSW
Williams 82	30.5±1.5	0.55±0.04	0.77±0.08	32.33±0.67	0.19±0.05	1.19±0.15
Yudou 29	39±2	0.56±0.11	1.16±0.09	27.5±16.5	0.51±0.14	0.83±0.4
PI437654	39±0	0.38±0.17	0.52±0.02	37.5±2.5	0.45±0.32	0.83±0.09
Zhonghuang 13	26.33±3.18	0.4±0.16	1.02±0.13	17±2	0.52±0.21	0.34±0.07
Forrest	26±1.58	0.42±0.15	0.48±0.07	31.67±3.33	0.22±0.05	0.84±0.1
Essex	32.5±2.5	0.58±0.01	0.68±0.08	28.33±3.53	0.27±0.15	0.44±0.12
JKD 2	20±5.51	0.37±0.05	0.96±0.12	16.33±6.98	0.43±0.16	0.86±0.28
Zhongdou 63	27.5±1.19	0.42±0.17	1.05±0.15	32.33±1.45	0.5±0.17	1.29±0.04
Zhongdou 57	29.33±2.33	0.58±0.24	0.81±0.07	36.5±3.5	0.28±0.07	0.74±0.11
Shendou 9	30±2.89	0.57±0.23	1.3±0.15	23.17±6.6	0.36±0.06	1.31±0.11
Yundou 1	35.5±3.04	0.46±0.11	1.11±0.32	25±7.02	0.62±0.24	1.32±0.21
Pudou 857	37.33±1.42	0.48±0.02	1.04±0.1	34.33±3.84	0.52±0.21	0.92±0.1
Loudou 1	36±2.86	0.23±0.07	0.63±0.08	23.83±7.34	0.3±0.1	0.93±0.11
Jiadou 2	33.33±1.67	0.2±0.04	1.11±0.2	31±1	0.52±0.15	1.33±0.18
Xiangxing 1	38±2.71	0.41±0.1	1.07±0.05	41.67±4.41	0.26±0.08	0.98±0.05
Liudou 99	35±0	0.29±0	1.18±0	43±7	0.18±0	0.87±0.27
Andou 203	35±2.04	0.49±0.14	1.45±0.19	29.5±2.36	0.34±0.04	1.25±0.05

Table S2-4. (Continued at 13th dpi).

Soybean Cultivar	Non-Inoculated Plants			Inoculated Plants		
	PH	FRW	FSW	PH	FRW	FSW
Williams 82	45.67±0.67	0.24±0.05	1.62±0.06	40.33±2.6	0.22±0.05	1.62±0.07
Yudou 29	48±2.52	0.33±0.08	1.39±0.27	44.67±1.45	0.24±0.03	1.46±0.08
PI437654	43.33±2.03	0.34±0.05	1.15±0.12	38±1.53	0.25±0.06	1.04±0.11

Zhonghuang 13	46±1	0.32±0.01	1.53±0.13	50.33±0.33	0.32±0.04	1.89±0.25
Forrest	44.67±2.4	0.24±0.1	1.11±0.05	38.33±4.91	0.21±0.04	1±0.08
Essex	34.67±2.4	0.16±0.04	0.75±0.06	36±3.06	0.19±0.06	0.72±0.01
JKD 2	46.67±1.67	0.29±0.05	1.55±0.17	42±2.08	0.31±0.04	1.85±0.16
Zhongdou 63	45.67±1.76	0.25±0.02	1.75±0.06	46±1	0.37±0.04	1.94±0.04
Zhongdou 57	44.67±0.88	0.3±0.04	2.13±0.14	38.5±6.75	0.57±0.23	1.57±0.17
Shendou 9	48.83±0.83	0.36±0.05	2.14±0.27	51±2.52	0.34±0.05	2.66±0.42
Yundou 1	45.33±2.91	0.39±0.02	2.6±0.29	49.33±0.33	0.31±0.07	2.91±0.16
Pudou 857	49.33±2.85	0.16±0.01	1.51±0.13	47±1.53	0.37±0.16	1.72±0.02
Loudou 1	47.67±1.45	0.37±0.07	1.85±0.22	47.67±1.33	0.17±0.04	1.64±0.19
Jiadou 2	45.67±0.33	0.25±0.04	1.92±0.21	49.67±0.33	0.24±0.02	2.31±0.24
Xiangxing 1	45.67±0.67	0.26±0.05	1.43±0.2	41±9.07	0.39±0.13	1.35±0.18
Liudou 99	43.33±2.33	0.2±0.04	1.54±0.29	46.33±1.33	0.27±0.12	1.54±0.3
Andou 203	49.33±0.67	0.29±0.06	1.72±0.04	50.33±0.33	0.26±0.06	2.07±0.38

Table S2-5. (Continued at 16th dpi).

Soybean Cultivar	Non-Inoculated Plants			Inoculated Plants		
	PH	FRW	FSW	PH	FRW	FSW
Williams 82	52±4.16	0.29±0.05	1.53±0.17	47.33±1.76	0.29±0.05	1.73±0.24
Yudou 29	65±2	0.38±0.06	1.72±0.23	56.33±3.18	0.34±0.06	1.78±0.18
PI437654	49±0.58	0.25±0.09	1.26±0.06	43±1.15	0.29±0.05	1.16±0.08
Zhonghuang 13	53±3	0.25±0.02	1.55±0.04	45.67±2.33	0.26±0.03	1.35±0.12
Forrest	52.33±1.2	0.23±0.07	1.24±0.04	34±1	0.16±0.02	0.87±0.05
Essex	38.33±0.67	0.21±0.05	0.92±0.06	36.33±3.53	0.24±0.07	0.82±0.02
JKD 2	55.33±4.67	0.27±0.06	1.51±0.26	41.67±1.67	0.25±0.05	1.21±0.25
Zhongdou 63	55±5.13	0.36±0.06	2.07±0.32	46±3.21	0.26±0.04	1.61±0.11
Zhongdou 57	49.33±0.67	0.26±0.03	1.76±0.15	52±3.61	0.29±0.06	2.08±0.15
Shendou 9	60.67±2.33	0.38±0.08	2.46±0.07	35±7.21	0.4±0.04	1.46±0.21
Yundou 1	55.33±2.03	0.26±0.08	1.98±0.3	52.33±0.88	0.25±0.06	2.07±0.06
Pudou 857	55.67±3.18	0.21±0.06	1.59±0.22	50.33±0.33	0.17±0.05	1.35±0.1
Loudou 1	52.67±3.76	0.21±0.04	1.55±0.36	55.33±3.71	0.14±0.05	1.71±0.23
Jiadou 2	54±3.06	0.34±0.1	2±0.23	41.33±11.29	0.3±0.11	1.72±0.13
Xiangxing 1	54±4	0.37±0.08	2.15±0.04	56.67±1.67	0.2±0.07	1.46±0.29
Liudou 99	52±5.69	0.39±0.16	1.58±0.21	49±3.21	0.16±0.06	1.51±0.24
Andou 203	58.67±3.18	0.19±0.07	1.39±0.16	46±2.08	0.22±0.08	1.21±0.28

Table S2-6. (Continued at 19th dpi).

Soybean Cultivar	Non-Inoculated Plants			Inoculated Plants		
	PH	FRW	FSW	PH	FRW	FSW
Williams 82	57.67±3.71	0.43±0.13	1.68±0.11	45.67±7.06	0.3±0.07	1.44±0.35
Yudou 29	62±4.36	0.36±0.01	1.65±0.17	51±4.93	0.33±0.03	1.6±0.26

PI437654	44.67±2.6	0.23±0.03	1.02±0.09	44.67±2.73	0.33±0.05	1.33±0.12
Zhonghuang 13	55.33±7.88	0.35±0.1	1.59±0.32	42.33±5.36	0.2±0.1	1±0.41
Forrest	53±2	0.25±0.01	1.17±0.07	38.33±2.03	0.23±0.01	0.85±0.13
Essex	38.33±3.33	0.23±0.01	0.76±0.02	38.33±1.2	0.24±0.07	0.74±0.05
JKD 2	53.67±3.67	0.27±0.01	1.65±0.23	28.33±6.33	0.33±0.07	1±0.18
Zhongdou 63	53.67±3.67	0.31±0.05	1.84±0.15	47.33±1.33	0.39±0.09	1.87±0.08
Zhongdou 57	52.33±1.45	0.49±0.06	2.1±0.2	50.33±4.67	0.4±0.12	1.9±0.46
Shendou 9	57.33±2.19	0.49±0.02	1.98±0.12	50±0.58	0.42±0.05	2.29±0.56
Yundou 1	58.33±6.64	0.39±0.07	2.61±0.34	55.67±3.48	0.23±0.05	2.12±0.29
Pudou 857	63±2.65	0.35±0.01	1.49±0.03	58.67±1.86	0.23±0.06	1.21±0.12
Loudou 1	53.67±2.91	0.42±0.08	1.78±0.3	58.67±5.78	0.18±0.04	1.8±0.44
Jiadou 2	60.33±0.33	0.51±0.16	2.16±0.17	63±3.51	0.27±0.08	2.42±0.2
Xiangxing 1	48.67±0.67	0.44±0.09	1.72±0.32	52.67±4.41	0.17±0.05	1.83±0.19
Liudou 99	60±2.65	0.32±0.04	1.68±0.24	50±5.77	0.26±0.11	1.51±0.39
Andou 203	67.67±1.76	0.42±0.09	2.3±0.35	52.67±5.04	0.24±0.09	1.42±0.3

Table S2-7. (Continued at 22nd dpi).

Soybean Cultivar	Non-Inoculated Plants			Inoculated Plants		
	PH	FRW	FSW	PH	FRW	FSW
Williams 82	67±2.52	0.29±0.01	1.42±0.22	56.25±4.03	0.36±0.01	1.48±0.12
Yudou 29	78±3.7	0.42±0.04	1.58±0.2	64.67±2.19	0.45±0.01	1.35±0.23
PI437654	73.67±18.44	0.29±0.01	1.32±0.27	57.33±8.29	0.27±0.02	1.19±0.16
Zhonghuang 13	68.33±3.71	0.26±0.01	1.13±0.11	69.33±10.73	0.35±0.01	1.48±0.21
Forrest	70±3.61	0.19±0.02	0.98±0.08	42.33±1.33	0.3±0.01	0.88±0.04
Essex	55±7.64	0.19±0.03	0.88±0.22	41.33±1.86	0.29±0.03	0.71±0.13
JKD 2	72.67±2.67	0.27±0.02	1.82±0.31	50.67±6.06	0.34±0.01	1.06±0.12
Zhongdou 63	64.67±8.95	0.25±0.02	1.61±0.16	58±2.52	0.55±0.06	2.12±0.22
Zhongdou 57	61.5±2.99	0.26±0.02	1.52±0.2	74.33±2.33	0.48±0.09	1.87±0.23
Shendou 9	69.33±6.36	0.37±0.02	1.6±0.35	78.5±7.89	0.46±0.07	2.27±0.15
Yundou 1	81.5±4.25	0.39±0.04	2.25±0.12	70±11.55	0.37±0.07	1.83±0.09
Pudou 857	83.5±3.07	0.28±0.04	1.43±0.1	57.67±3.84	0.3±0.07	1.19±0.15

Loudou 1	76.33±4.84	0.27±0.01	1.71±0.17	77.5±7.5	0.25±0.04	1.89±0.37
Jiadou 2	68.33±13.17	0.43±0.04	2.02±0.22	89±6.66	0.37±0.05	2.26±0.13
Xiangxing 1	69.33±7.22	0.28±0.01	1.49±0.27	84.75±5.36	0.31±0.02	1.52±0.09
Liudou 99	66.67±7.26	0.23±0.02	1.12±0.28	58.25±4.8	0.25±0.04	1.18±0.28
Andou 203	70.25±5.31	0.35±0.04	1.14±0.11	85±6.45	0.3±0.02	1.74±0.17

Table S2-8. (Continued at 25th dpi).

Soybean Cultivar	Non-Inoculated Plants			Inoculated Plants		
	PH	FRW	FSW	PH	FRW	FSW
Williams 82	88.33±14.81	0.35±0.06	1.68±0.31	41.67±4.41	0.25±0.03	0.88±0.25
Yudou 29	70±14.42	0.33±0.02	1.21±0.07	78.67±1.33	0.32±0.02	1.71±0.28
PI437654	66.67±6.67	0.28±0.04	1.14±0.09	55.67±3.48	0.25±0.06	1.04±0.06
Zhonghuang 13	93.33±4.37	0.39±0.08	1.56±0.45	51.67±6.01	0.18±0.09	1.05±0.48
Forrest	74.67±4.33	0.2±0.01	0.81±0.08	46.33±4.18	0.25±0.06	0.93±0.11
Essex	52±6.08	0.17±0.02	0.83±0.07	44.67±8.01	0.3±0.02	0.65±0.12
JKD 2	61.67±14.24	0.29±0.09	1.36±0.27	34.67±3.18	0.25±0.03	0.86±0.05
Zhongdou 63	86.33±8.57	0.35±0.04	1.65±0.28	66±3.21	0.41±0.07	1.69±0.45
Zhongdou 57	78.67±5.78	0.42±0.07	1.14±0.35	94.33±16.6	0.35±0.06	1.96±0.2
Shendou 9	146.67±20.28	0.47±0.04	3.08±0.1	81±15.31	0.38±0.02	1.73±0.13
Yundou 1	96±5.86	0.55±0.13	2.24±0.38	88.67±17.37	0.3±0.02	2.14±0.36
Pudou 857	92±10.6	0.35±0.02	1.3±0.02	72.67±18.75	0.27±0.04	1.41±0.24
Loudou 1	94.67±7.75	0.39±0.05	2.02±0.13	57.33±8.76	0.28±0.05	1.36±0.16
Jiadou 2	81.33±1.86	0.44±0.11	2.07±0.21	63.33±3.33	0.23±0.03	1.72±0.06
Xiangxing 1	86.67±4.41	0.39±0.06	1.57±0.13	82.67±0.33	0.26±0.03	1.03±0.06
Liudou 99	79.33±4.7	0.22±0.04	2.29±0.68	53±9.54	0.19±0.04	1.05±0.18

Andou 203	88.67±11.41	0.35±0.04	1.4±0.3	73±6.51	0.27±0.01	1.36±0.18

Table S2-9. (Continued at 28th dpi).

Soybean Cultivar	Non-Inoculated Plants			Inoculated Plants		
	PH	FRW	FSW	PH	FRW	FSW
Williams 82	96.33±12.55	0.4±0.02	1.91±0.15	43.33±1.67	0.13±0.04	1.25±0.18
Yudou 29	105.67±9.82	0.52±0.08	1.9±0.12	73.67±2.33	0.33±0.05	1.32±0.04
PI437654	100±13.23	0.37±0.01	1.57±0.25	51±0.58	0.3±0.11	0.99±0.08
Zhonghuang 13	75.67±3.84	0.29±0.03	1.39±0.2	63.67±5.84	0.26±0.03	1.11±0.08
Forrest	93±7	0.25±0.01	1.21±0.14	42±4.36	0.4±0.06	0.79±0.04
Essex	69.67±11.55	0.22±0.02	1.01±0.25	38.67±3.67	0.23±0.04	0.61±0.04
JKD 2	78.33±9.24	0.35±0.05	1.72±0.23	44.67±14.62	0.36±0.03	1.35±0.01
Zhongdou 63	122.67±5.04	0.36±0.05	2.78±0.31	86.67±4.41	0.44±0.06	1.79±0.1
Zhongdou 57	75±2.89	0.47±0.13	1.44±0.11	82.33±11.78	0.39±0.06	1.69±0.17
Shendou 9	110±12.58	0.39±0.01	2.65±0.08	91.67±9.28	0.43±0.05	2.06±0.1
Yundou 1	106±4.93	0.45±0.03	2.37±0.16	86.67±6.69	0.36±0.04	1.99±0.17
Pudou 857	77.5±2.5	0.23±0.05	1.37±0.23	72.33±9.39	0.24±0.02	1.35±0.09
Loudou 1	108.33±6.89	0.34±0.01	2.11±0.17	78±9.64	0.15±0	1.07±0.11
Jiadou 2	89.67±5.7	0.37±0.02	1.96±0.36	78.33±12.02	0.33±0.06	1.56±0.28
Xiangxing 1	102.33±1.45	0.35±0.03	1.76±0.09	94.67±5.17	0.28±0.06	1.42±0.2
Liudou 99	90±2.89	0.23±0.06	1.92±0.36	46.33±6.33	0.25±0.04	1.14±0.25
Andou 203	87±3.51	0.43±0.1	1.38±0.09	80±11.55	0.26±0.01	1.6±0.16

Table S2-10. (Continued at 45th dpi).

Soybean Cultivar	Non-Inoculated Plants			Inoculated Plants		
	PH	FRW	FSW	PH	FRW	FSW
Williams 82	152.67±24.11	1.86±0.12	3.49±0.6	42.33±8.19	0.49±0.01	0.72±0.17
Yudou 29	127.14±13.4	0.61±0.07	1.77±0.24	82.29±4.06	0.52±0.04	1.39±0.13
PI437654	95.38±10.06	0.7±0.14	1.55±0.14	64.5±5.54	0.35±0.04	1.21±0.1
Zhonghuang 13	66.67±6.67	0.26±0.07	1.19±0.06	59±6.66	0.14±0.1	0.5±0.33
Forrest	69±0	0.52±0	1.22±0	67.67±9.6	0.47±0.05	0.97±0.14
Essex	83.5±18.77	0.69±0.26	1.58±0.54	63.75±4.75	0.31±0.01	0.75±0.07
JKD 2	104±6.57	1.32±0.18	2.38±0.18	66.2±5.13	0.4±0.04	1.36±0.1
Zhongdou 63	148.67±9.77	2.69±0.45	2.95±0.59	110±16.17	0.71±0.02	1.89±0.34

Zhongdou 57	91.57±5.27	0.54±0.04	1.57±0.18	77.14±6.53	0.53±0.03	1.7±0.21
Shendou 9	119±0	0.64±0	2.16±0	116.33±28.42	0.59±0.08	2.65±0.62
Yundou 1	142.33±25.46	0.94±0.19	1.56±0.18	133.33±32.83	0.44±0.13	1.85±0.43
Pudou 857	80±0	0.43±0	1.6±0	75±13.65	0.31±0.01	1.38±0.25
Loudou 1	123.17±5.84	0.55±0.1	1.61±0.26	108.33±13.48	0.61±0.16	1.63±0.34
Jiadou 2	88.33±7.26	0.56±0.09	1.64±0.15	113.5±17.25	0.42±0.07	2.23±0.28
Xiangxing 1	127.5±1.89	0.59±0.24	0.94±0.25	117.25±19.84	0.47±0.07	1.83±0.36
Liudou 99	107.13±8.92	0.48±0.04	1.67±0.14	82.5±12.5	0.43±0.03	1.59±0.21
Andou 203	85.33±2.91	0.45±0.02	1.19±0.06	112.67±21.61	0.5±0.07	1.87±0.13

Table S2-11. (Continued at 60th dpi).

Soybean Cultivar	Non-Inoculated Plants			Inoculated Plants		
	PH	FRW	FSW	PH	FRW	FSW
Williams 82	90±0	0.14±0	0.55±0	97±19.14	0.23±0.07	0.94±0.26
Yudou 29	164.67±18.81	0.65±0.03	1.62±0.4	154.67±24.5	0.55±0.05	1.66±0.23
PI437654	130±10	0.49±0.02	0.85±0.31	163±15.62	0.46±0.13	1.6±0.15
Zhonghuang 13	130±0	0.36±0	1.17±0	141.33±27.72	0.38±0.03	1.39±0.5
Forrest	170±0	0.41±0	1.01±0	133.33±29.54	0.3±0.03	1.42±0.11
Essex	85.33±17.75	0.14±0.02	0.51±0.08	140.5±18.7	0.35±0.05	1.38±0.37
JKD 2	173±0	0.49±0	1.59±0	60.75±15.18	0.34±0.04	1.2±0.3
Zhongdou 63	134.75±19.59	0.76±0.14	1.78±0.3	134±25.38	0.33±0.1	1.8±0.38
Zhongdou 57	127.4±17.94	0.95±0.14	1.62±0.37	157.2±16.3	0.78±0.12	2.22±0.21
Shendou 9	162.33±13.67	0.49±0.01	1.63±0.21	209±39.55	0.77±0.13	3.06±0.4
Yundou 1	140±32.62	0.53±0.07	1.47±0.27	165.33±8.67	0.47±0.09	2.07±0.19
Pudou 857	128±0	0.56±0	0.97±0	130±27.54	0.2±0.05	0.81±0.29
Loudou 1	152±0	0.26±0	1.9±0	144.33±20.22	0.4±0.06	1.74±0.33
Jiadou 2	207±0	0.51±0	2.31±0	191.67±27.74	0.58±0.1	2.38±0.39

Xiangxing 1	210±0	0.89±0	2.51±0	159.67±19.92	0.62±0.12	2.09±0.5
Liudou 99	151.33±24.31	0.41±0.02	1.43±0.17	128.33±43.43	0.44±0.16	1.46±0.57
Andou 203	103.33±13.33	0.34±0.01	1.44±0.18	186.67±34.68	0.62±0.21	2.45±0.72

Note： Values are the means of seven replicates ± SE.
